# Uygur Medicine Xipayi Kui Jie’an Affects Gene Expression profiles in intestinal tissue Lesions in a Rat Model of Ulcerative Colitis

**DOI:** 10.1186/s12906-015-0672-x

**Published:** 2015-05-22

**Authors:** Kurexi Yunusi, Jingping Zhang, Li Zhong, Gulinisha Mosha, Ajiguli Nuermaimaiti, Mairipaiti Abudula, Halmurat Upur

**Affiliations:** Department of Molecular Biology and Biochemistry, College of Basic Medicine, Xinjiang Medical University, Urumqi, Xinjiang 830000 China; Traditional Uighur Medicine Institute, Xinjiang Medical University, Urumqi, Xinjiang 830000 China

**Keywords:** Uygur Medicine, Ulcerative colitis, Xipayi kuijie’an, Immunity and inflammation, Microarray

## Abstract

**Background:**

The aim of this study was to investigate the mechanisms underlying the therapeutic effect of Uygur medicine KJA on UC in a rat model.

**Methods:**

UC was induced in Wistar rats by application of 2, 4-dinitrochlorobenzene and acetic acid and were then treated with three different doses of KJA, and normal saline as control. After treatment for 20 days, the gene expression profile of colonic tissue was analyzed by microarray and verified by quantitative real-time RT-PCR.

**Results:**

Animals treated with the three different doses of KJA were compared with normal saline controls, wherein microarray analysis identified 1991, 2163, and 1677 differentially expressed genes respectively, of which 444 genes were raised and 670 genes were decrease spliced together in the three doses tested. The KEGG pathway analyses found commonly raised genes related to several different biological functions. Interesting genes included TRL2, IL-1β, TGF-β1, and NF-κB were confirmed by quantitative PCR.

**Conclusions:**

The therapeutic effect of KJA on UC is likely explained by specific effects on the expression of genes, which are the effector molecules known to be involved in the development of UC. Further studies on differentially expressed genes will help explain the mechanism of action of Uygur medicine KJA.

## Background

Ulcerative colitis (UC) is a chronic non-specific inflammation and ulceration of the digestive tract, involving mainly mucous membranes of the large intestine with serious complications and often recurrent attacks lasting several months to years [1]. UC is common in the rectum, sigmoid flexure and left colon. The incidence of UC is in the range of approximately 0.5 – 24.5/100,000 of the population depending upon the economic status of the country [2, 3]. It is classified as one of the refractory diseases by WHO [4].

Although there has been marked progress in understanding the pathogenesis of the disease over the last several years, the main causes of UC remain unclear. Environmental factors, immunologic factors, and genetic susceptibility seem to partly contribute to the development of chronic inflammation in the gut [5–9]. Currently, there is no curative treatment available for UC. Various drugs that are used systemically and/or topically to induce and maintain remission include aminosali cylates, glucocorticosteroids and immunomodulators [10–16]. Surgical therapy is an option for patients with toxic colonitis who depend on prolonged hormonal therapy. However, surgical therapy requires resecting the entire intestine and inosculating ileum and anus; however, about 40% of patients appear with acute inflammation of the bursa bag [17]. Patients without proper treatment may develop colon cancer [18, 19]. Therefore, novel and effective therapeutics exhibiting reduced toxicity are urgently needed for treating UC.

Uygur Medicine is an ethnic medical system, and broadly categorized as alternative medicine. Some of the drugs commonly prescribed in Uygur Medicine have shown promising therapeutic effects in UC. Uygur Medicine Xipayi Kui Jie’an (KJA) prescription from the uygur medicine approach called “Xipayi gingiva protective solution”, in accordance with the Ministry of Health of the People’s Republic of China Pharmaceutical Standards – Uighur Medicine [20] from the 1998 edition, is composed of gallic acid. Previously we have reported that KJA is curative in a rat model of UC by colon tissue morphology and pathological changes. In this model, we observed reduced inflammation, and evaluated the scope of curative effect after treatment [21–23]. To explore the potential mechanisms of the therapeutic effect of KJA on UC, we used gene chip technology and examined the effect of KJA on gene expression profiles of diseased intestinal tissues from UC rats.

## Methods

### Experimental animals

Specific pathogen-free (SPF) adult male Wistar rats (220-250 g) were provided by the Experimental Animal Center of Xinjiang Medical University. They were housed in a controlled environment (temperature 24–25°C, humidity 70%–75%) and were fed on a normal laboratory diet. Rats were rested for at least 1 week before use. All experiments were conducted according to the guidelines of the local ethics committee at Xinjiang Medical University (Permit Number: IACUC-20121122011).

### Establishment of UC rat model

Experimental animal grouping consisted of 109 Wistar rats that were stochastically divided into the following groups: normal group (7 rats); an ulcerative colitis model group (21 rats with 5 deaths); a physiological saline/negative control group (21 rats, 10 deaths); the Xipayi Kui Jie’an large dose intervention group (19 rats, 7 deaths); the medium dose intervention group (20 rats, 5 deaths); and the small dose intervention group (21 rats, 5 deaths). We created a rat model of UC using 2.4-dinitrochlorobenzene (DNCB) composite in acetic acid. The rats were administered 0.25 mL of DNCB acetone liquid (DNCB 2.0 g:acetone 100 mL) solution on depilated areas of the back and neck daily for 14 days. The DNCB, acetone, and ethanoic acid used in this experiment were domestically produced and analytically pure. Following application of the DNCB on day 14, the rats were fasted (with free access to water drinking) for 24 h. On day 15, 0.25 mL of a 0.1% DNCB ethyl alcohol solution was injected into the rat colon via a 3 mm-diameter catheter that was inserted 8 cm past the anus. On day 16, the same procedure was used to inject 2 mL of 6% ethanoic acid. The solution was allowed to have an effect for 15 s, before 5 mL of physiologic saline solution was used to flush the area. Subsequently, rats were fed for 7 days and stool characters, dietetics, hair condition and activity were monitored daily. Observations showed that that the rats gradually produced typical symptoms of UC, thus confirming that the model of UC was successfully established. The normal group rats fed without any processing.

### Source of drugs

Xipayi Kui Jie’an Enema (Preparation Standard of Medical Institutions Formulated by the Food and Drug Administration of Xinjiang Uygur Autonomous Region ZZJ-0001-2013) is composed of garlic (liquid herbal extract, 50 mL/bag includes 2 g of crude drug), and was provided by Xinjiang Cicon Habo Uyghur Medicine Co., Ltd. (Urumqi, China). The common therapeutic dose of KJA for humans is 23 mg/kg/d. The equivalent dose in rats is 150 mg/kg/d; this was used as the medium dose, while 75 mg/kg/d and 300 mg/kg/d were used as the low and high doses, respectively.

[Preparation method]: The medicinal material nutgall was taken and crushed into coarse powders. Water 10 times the amount was added for 8 h of overnight soaking. Then after the material was decocted for 3 times with 1 h each time, the decoctions were combined and pressured with the plate and frame (0.3 MPa). After that, the combined decoction was filtrated with a speed of 5 tons/h (200 mesh filter cloth) and the filtrate was added with 0.2% sodium benzoate which was dissolved by stirring to prepare 1000 ml liquid. Finally, the enema was obtained after repackaging.

[Identification]: 1 ml of the product was taken and added with water to 10 ml. Then ethyl acetate was added and extracted 2 times after vibration with 5 ml each time. The ethyl acetate liquid was combined and dried. And the residual was added with 2 ml methanol for dissolution and used as the test solution. Gallic acid was additionally taken as the control and added with methyl alcohol to be the solution with 1 mg Gallic acid in 1 ml solution as the control. According to the test of thin layer chromatography (TLC) (appendix VI B of Chinese pharmacopoeia, 2010 edition), 5 μl control solution and 2 μl test solution were absorbed and respectively dripped on the same thin layer plate made up of silica G with sodium carboxymethyl cellulose as the binder. And after they were developed with chloroform-ethyl acetate-formic acid (6:4:1) as the developer, they were taken out, dried in the air and sprayed with 1% ferric chloride ethanol solution. In the chromatogram of the test solution, spots were shown with the same color at the corresponding positions of the chromatography of the control solution.

### Treatment of animals

The rats with treatment were clystered using a suitable tube inserted into the colondaily with normal saline (22.5 mL/kg) as a negative control, and with low, medium and high doses of KJA, should choose liquid paraffin wax was used as a lubricant. After 20 days of treatment, the animals were fasted overnight before being sacrificed by administering an overdose of ether . The distal colon with a length of 5-8 cm was removed for extraction of total RNA. After removing the connective and adipose tissues, the colon was cut open along the mesenteric edge and rinsed from the inside gently with cold RNase-free saline. The colon tissue samples were stored at -80°C before isolation of total RNA.

### Transcriptomic analysis

The total RNA was extracted from the colon tissues by the Trizol (Invitrogen, Gaithersburg, MD, USA) one-step method. After enrichment of RNA by isopropyl alcohol precipitation, total RNA was column purified by using a NucleoSpin® RNA clean-up kit (MACHEREY-NAGEL, Germany). The concentration and purity of total RNA were determined by a spectrophotometer, and the quality assessment was conducted by the integrity of 28S and 18S rRNA. We used the rat genome 70-mer oligonucleotide microarray version 3.05, representing about 22, 012 genes and 27, 044 transcripts (for detailed information refer to http://www.Operon.com), from the CapitalBio Corporation (Beijing, China) [24, 25] for monitoring changes in gene expression profiles.

### Microarray imaging and data analysis

Arrays were scanned with a confocal LuxScan^™^ scanner and the images were analyzed using LuxScan^™^ 3.0 software (both from CapitalBio). For individual channel data extraction, faint spots with intensities below 400 units after background subtraction in both channels (Cy3 and Cy5) were removed and a space- and intensity-dependent normalization that was based on a LOWESS program was applied [26]. The significance of differentially expressed genes was determined using Significance Analysis of Microarrays (version 3.02). We set the criteria for differentially expressed genes as the ratio of two channels for the same gene as ≥ 2 or ≤ 0.5, P < 0.05, after allowing for quality control analysis and normalization of the data.

### Bioinformatics analysis of differentially expressed genes

Functional analysis of gene expression was performed using CapitalBio Corporation Bio-molecular function annotation system (MAS system http://www.capitalbio.com) to annotate the differentially expressed genes and permit statistical analysis of the pathway enrichment. The CapitalBio Molecule Annotation System (MAS; version 4.0), KEGG, and GenMAPP were used for pathway analysis (http://bioinfo.capitalbio.com/mas) [27]. For each pathway, genes with known rat orthologues were compared with sets of significant genes from SAM to define the effects of the corresponding pathway.

### Confirmation of microarray results with quantitative real-time RT- PCR

A Crystal core® EvaGreen fluorescence quantitative two-step RT-PCR universal kit (CapitalBio) was used to verify the differentially expressed genes that were identified by microarray analysis. Primer sequences (see Table 1) for amplification of TLR2, IL-1β,NF-κB, and TGF-β1 were designed based on the sequences published in GeneBank using the software program Primer Premier Express v5.0, and synthesized by the Takara Company (Dalian ,china). The PCR amplification was conducted at 95°C for 15 min, followed by 40 cycles of 94°C for 5 s, 58°C for 15 s, and 72°C for 10 s. The changes in target genes were determined using the 2^−ΔΔCt^ method [28].

**Table 1:** Sequences of primers used in quantitative real-time reverse transcription polymerase chain reaction

Gene symbol	Primer Sequences	Length
TLR2	Forward:5′-CTTACAGGACACTGGGGGAAC-3′	134bp
	Reverse:5′-AAGTTCGTTGAGAGAGGTCAGC-3′	
NF-κB	Forward:5′-TGATGACATACTCCCACAAG-3′	145 bp
	Reverse:5′-CAATATCCCCAGACCTAAC-3′	
IL-1β	Forward:5′-GACCTGTTCTTTGAGGCTGAC-3′,	578 bp
	Reverse:5′-TCCATCTTCTTCTTTGGGTATTGTT-3′	
TGF-β1	Forward:5′-GAGAGCCCTGGATACCAACTA-3′	173 bp
	Reverse:5′-CTGGTGTGTCCAGGCTCCAAATGT-3′	
β-actin	Forward:5′-AACTCCATCATGAAGTGTGA-3′	248 bp
	Reverse:5′-ACTCCTGCTTGCTGATCCAC-3′	

### Statistical analysis

Data analysis was performed using SPSS software v13.0. Values were reported as mean ± SD. ANOVA followed by Student’s *t*-test was used for multiple comparisons of the data. Statistical significance was set at an alpha value of p < 0.05.

## Results

### The effect of KJA on the gene expression profile in intestinal mucosa in rats with UC

To understand the underlying mechanism of KJA to UC rats, we established a rat UC model and examined the effect of KJA treatment on the gene expression profile in local colon tissues by cDNA microarray analysis. Compared to the three different doses (high, medium, and low) of KJA with normal saline control rats, the microarray analysis identified 1991, 2163, and 1677 differentially expressed genes in the rat respectively, of which 444 genes were raised and 670 genes were decrease spliced together in the three doses tested . Genes that showed significant changes in expression were mapped to the KEGG pathways (Tables 2, and 3). Commonly raised genes were related to oxidative phosphorylation, disease, and metabolism reactions. Cut genes related to cell adhesion molecules (CAMs), immune responses, metabolism, and signaling pathways. This suggested the involvement of complicated mechanisms in the effect of KJA to UC

**Table 2:** Significantly Changed Genes (different doses common raised) Mapped to KEGG Pathways

Pathway	Count	p-Value	q-Value	Gene
Cell adhesion molecules (CAMs)	13	9.35E-12	6.95E-11	Cdh1;RT1-N3;Cd8a;Jam3;Itgb7;Cd274;Cldn5;Cd8b;
				Icam2;H2-T24;Itgam;Itgb2;Cldn4
Natural killer cell mediated cytotoxicity	12	8.04E-11	3.78E-10	Rac2;RT1-N3;Ptpn6;Lck;Grb2;Zap70;Icam2;H2-T24;
				Tyrobp;Fcgr3;Itgb2;Prf1
Focal adhesion	13	1.61E-10	6.43E-10	Rac2;Fn1;Col5a1;Grb2;Itgb7;Fn1;Col1a2;Col1a1;
				Col4a2;Col1a1;Col4a1;Tnn;Flna;Ccnd1;Myl9
Complement and coagulation cascades	9	3.42E-10	1.27E-09	C1qb;C4b
				;C1qa;C1r
				;C1s;Cfb;
				C2;C6;Plau
ECM-receptor interaction	8	1.55E-08	3.84E-08	Fn1;Col5a1;Itgb7;Fn1;Col1a2;Col1a1;Col4a2;
				Col1a1;Col4a1;Tnn
Leukocyte transendothelial migration	9	4.12E-08	8.92E-08	Rac2;Jam3;Cldn5;Thy1;Itgam;Itgb2;Cxcr4;Myl9;Cldn4
Primary immunodeficiency	6	6.53E-08	1.36E-07	Cd8a;Ciita;Lck;Zap70;Rfxap;Cd8b
Arachidonic acid metabolism	6	1.61E-06	2.61E-06	Ephx2;Cyp2j4;Gpx3;Ptgs1;Pla2g10;Cbr1
Systemic lupus erythematosus	8	2.56E-06	4.04E-06	C1qb;C4b;C1qa;C1r;C1s;C2;C6;Fcgr3
Antigen processing and presentation	7	2.92E-06	4.22E-06	RT1-N3;Cd8a;Ciita;Rfxap;Cd8b;H2-T24;Cd74
Regulation of actin cytoskeleton	9	5.00E-06	7.03E-06	Cfl1;LOC688430;Rac2;Fn1;Pip4k2a;Itgb7;Fn1;
				Itgam;Itgb2;Myl9
T cell receptor signaling pathway	6	6.53E-05	7.54E-05	Cd8a;Ptpn6;Lck;Grb2;Zap70;Cd8b
Hematopoietic cell lineage	5	1.02E-04	1.09E-04	Cd8a;Csf1r;Anpep;Cd8b;Itgam
Axon guidance	6	1.25E-04	1.30E-04	Cfl1;LOC688430;Rac2;Slit3;Fes;Cxcr4
Small cell lung cancer	5	2.92E-04	2.62E-04	Fn1;Nos3;Fn1;Col4a2;Col4a1;Ccnd1
Citrate cycle (TCA cycle)	3	7.92E-04	6.44E-04	Pck1;Aco1;Acly

**Table 3:** Significantly Changed Genes (different doses cut together) Mapped to KEGG Pathways

Pathway	Count	p-Value	q-Value	Gene
Oxidative phosphorylation	24	4.93E-28	5.32E-26	Atp5b;Cox4i1;Ndufb5;Cox6c;Cox5a;LOC685596;Cox6c1;Atp5a1;LOC683884;Ndufab1;Cox5b;Cox6b1;RGD1559626;Atp5h;Ndufa5;Uqcrfs1;Ndufb8;Ndufa3;LOC684509;Atp5f1;LOC692052;LOC679739;Uqcrq;Ndufs5
Alzheimer's disease	27	2.22E-27	1.20E-25	Atp5b;Cox4i1;Plcb3;Ndufb5;Cox6c;Cox5a;Plcb4;LOC685596;Cox6c1;Atp5a1;LOC683884;Ndufab1;Cox5b;Cox6b1;RGD1559626;Atp5h;Ndufa5;Uqcrfs1;Ndufb8;Ndufa3;LOC684509;Atp5f1;LOC692052;LOC679739;Uqcrq;Ndufs5;Lpl
Parkinson's disease	24	8.34E-27	3.00E-25	Atp5b;Cox4i1;Ndufb5;Cox6c;Cox5a;LOC685596;Cox6c1;Atp5a1;LOC683884;Ndufab1;Cox5b;Cox6b1;RGD1559626;Atp5h;Ndufa5;Uqcrfs1;Ndufb8;Ndufa3;LOC684509;Atp5f1;LOC692052;LOC679739;Uqcrq;Ndufs5
PPAR signaling pathway	9	1.49E-10	1.46E-09	Fabp2;Hmgcs2;Ehhadh;Acadm;Cpt1a;Pdpk1;Cpt2;Lpl;Angptl4
Valine, leucine and isoleucine degradation	7	3.94E-09	3.54E-08	Hmgcs2;Ehhadh;Acadm;Acaa2;Pccb;Acaa2;Hadha;Bcat1
Fatty acid metabolism	6	7.74E-08	5.57E-07	Ehhadh;Acadm;Acaa2;Cpt1a;Acaa2;Hadha;Cpt2
Arginine and proline metabolism	5	7.00E-07	4.45E-06	Ckmt1;Ckb;Eprs;Rars;P4ha3
Drug metabolism - other enzymes	5	5.56E-06	2.73E-05	Ugt1a3;Ugt1a6;Ugt1a1;Ces2;Upp1
Fatty acid elongation in mitochondria	3	1.69E-05	7.95E-05	Acaa2;Acaa2;Hadha;Ppt1
Propanoate metabolism	4	2.12E-05	9.55E-05	Ehhadh;Acadm;Pccb;Hadha
Drug metabolism - cytochrome P450	5	2.89E-05	1.20E-04	Ugt1a3;Ugt1a6;Ugt1a1;Gstm5;Fmo1

### Real-time fluorescence quantitative RT - PCR analysis of differentially expressed genes

**Fig. 1:**
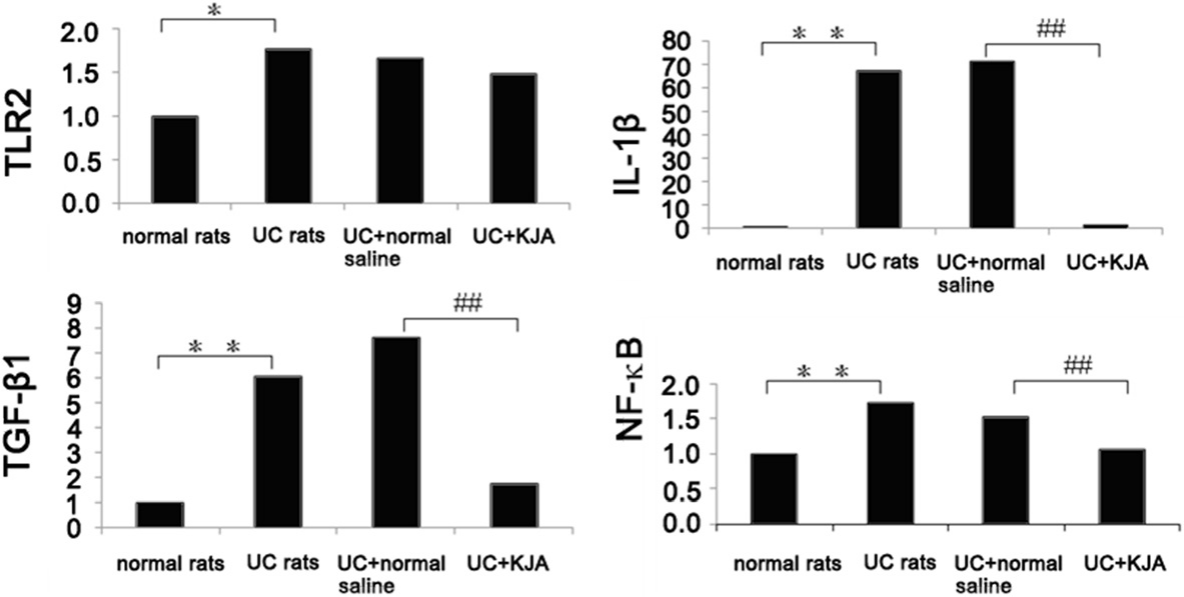
Quantitative real-time RT-PCR analysis of the selected gene expression in rat colon tissue. The total RNA samples from normal rats, UC rats, UC rats treated with normal saline (UC + normal saline) and UC rats that were treated with low dose KJA (UC + KJA) were extracted and mRNA expression levels for TLR2, IL-1β, TGF-β1 and NF-κB were quantified using β-actin as an internal control for normalization. Means and SD from 3 rats per group are shown. UC rats vs. normal rats: * P < 0.05,and ** P < 0.01; UC + KJA vs. UC + normal saline: #P < 0.05,and ##P < 0.01

**Fig. 2:**
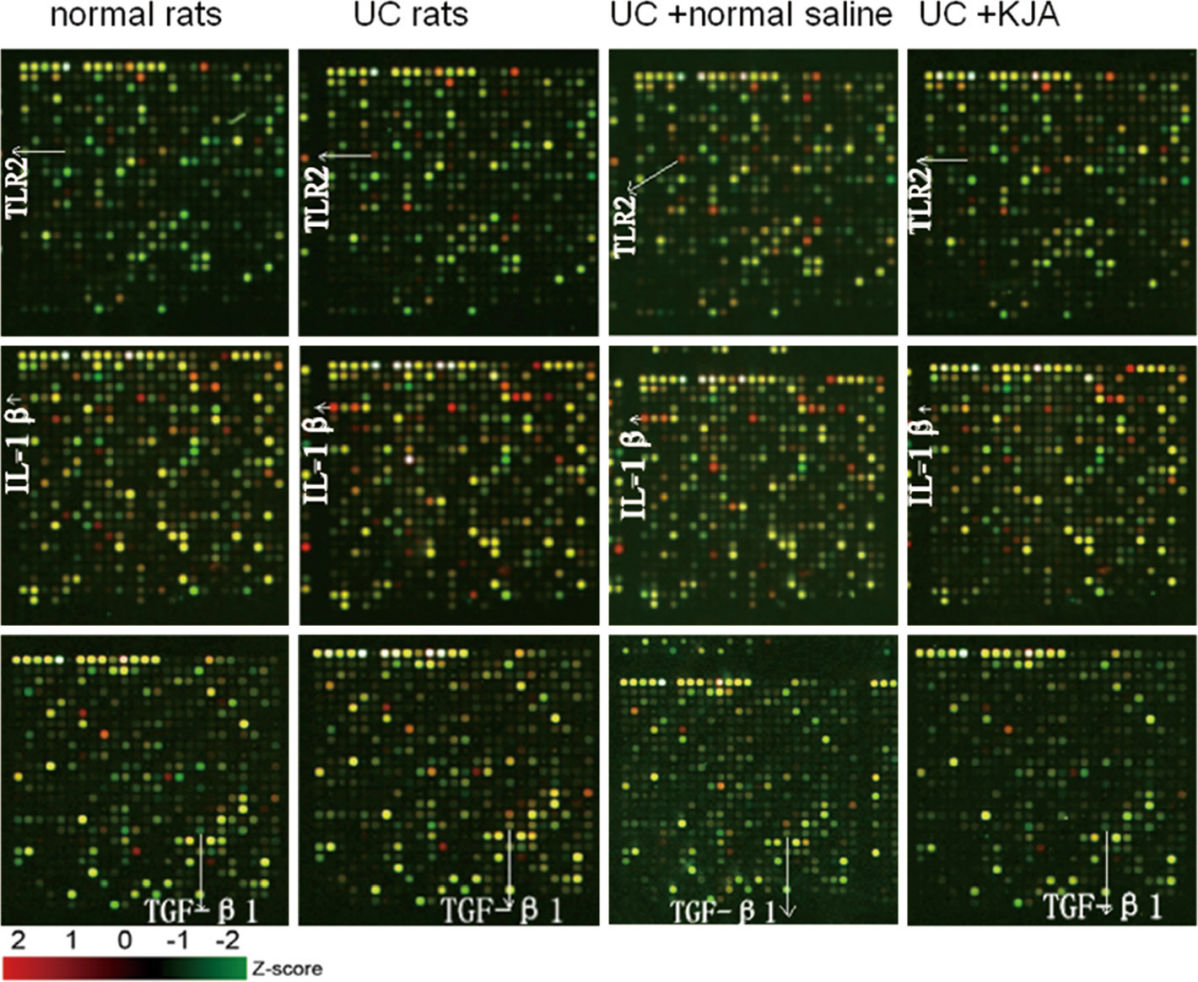
The effect of KJA on TLR2, IL-1β and TGF-β1 expression in colon tissues from rats. Representative microarray images of cDNA samples derived from different groups of rats are shown (n = 3). The arrows indicate the corresponding gene spots for TLR2, IL-1β and TGF-β1. Red color denotes an increase of gene expression, whereas green colored spots indicates a decrease

After identification of differentially expressed genes by KJA treatment by microarray analysis, we performed quantitative RT-PCR for selected genes in the intestinal samples from UC rats treated with KJA to confirm the microarray data. For this, we concentrated on evaluation of TRL2, IL-1β, TGF-β1, and NF-κB, since these genes were implicated in the development of UC [29, 30]. We evaluated the expression of these genes using quantitative real-time RT-PCR from total RNA samples isolated from 3 rats representing each group. Our results demonstrated that expression of these genes were increased significantly in intestinal mucosa of UC rats as compared with that expressed in the intestinal mucosa of normal rats (Fig. 1). Treatment of UC rats with KJA for 20 days differentially reduced the expression of IL-1β, TGF-β1, TLR2 and NF-κB in the low dose group as compared with the normal saline control group. These results were consistent with the microarray data (Fig. 2). In conclusion, our studies with quantitative PCR analysis of selected genes identified with microarray analysis further verified differentially expressed genes in rats with UC as compared with that found in normal rats. Our observations also demonstrated the specific effect of KJA treatment on the expression of these genes in rats with UC. Thus, we conclude that KJA exerts its therapeutic effects by differentially regulating expression of several different key molecules associated with the development of UC.

## Discussion

Ulcerative colitis is a chronic inflammatory bowel disease that predominantly involves the large intestine with limited therapeutic options, with the notable exception of continuous application of anti-inflammatory reagents including corticosteroids. In the current report, we showed induction of UC in rats following the application of acetic acid combined with DNCB, a commonly used animal model of UC [31], and studied the effects of applying Uygur medicine KJA in rats with UC. The changes in gene expression patterns were seen at all three doses of KJA, and these results suggest that application of KJA in UC rats induced significant changes in genes expression, and provided a potential mechanism for the therapeutic effect of KJA on UC. Treatment of UC rats with three different concentrations of KJA for 20 days changed the expression profile of these genes in the intestinal tissues as compared with UC rats treated with normal saline. We concluded all changes gene simultaneously in all three doses of KJA. The differentially expressed genes represent several different functional groups, and are mainly involved in oxidative phosphorylation, cell metabolism, cell signaling, and inflammation and immunity. The increase in expression of genes involved in “cell adhesion molecules (CAMs)” and “regulation of actin cytoskeleton” indicates that KJA may play a significant role in control of both the actin cytoskeleton and adhesion in the process of mucosal repair. Expression of genes involved in “natural killer cell mediated cytotoxicity” and “T cell receptor signaling pathway” functions suggested that an autoimmune response is involved in the treatment of UC with KJA. On the other hand, the decrease in the expression of genes involved in “oxidative phosphorylation”, “Alzheimer’s disease” and “Parkinson’s disease” indicates that KJA contributes to the risk of mitochondrial DNA variants. A significant decrease in expression of genes involved in “valine, leucine and isoleucine degradation”, “fatty acid metabolism”, “arginine and proline metabolism”, “propanoate metabolism” and increased expression of genes involved in the “citrate cycle” indicate that KJA decreases protein and lipid metabolism and increases the energy status of the citrate cycle, thereby regulating metabolism during chronic inflammation.

Our studies identified the gene expression patterns that were associated with immunity and inflammatory reactions, including TLR2, NF-κB, IL-1β and TGF-β1, with quantitative PCR analysis in the normal rat group, UC rat group, normal saline control group, and KJA small dose group respectively.

TLR2 is a member of the Toll-like receptor (TLR) family, and TLRs play fundamental roles in pathogen recognition and activation of innate immunity. The various TLRs exhibit different patterns of expression. TLR2 is expressed most abundantly in peripheral blood leukocytes, and mediates host immune responses to Gram-positive bacteria and yeast via stimulation of NF-κB [32–34]. In normal intestine, TLR2 is expressed mainly in lamina propria mononuclear cells and intestinal epithelial cells at low levels. Increased expression of TLR2 in patients with UC is caused mainly by increased leukocyte infiltration in the intestinal mucosa, which has been associated with the development of UC [35]. IL-1β is also shown to mediate the pathogenesis of UC [30]. The most significant and relevant properties of IL-1β are the initiation of cyclooxygenase type 2, inducible nitric oxide synthase and phospholipase A2, which are produced by various cell types [36]. TGF-β1 belongs to transforming growth factor beta (TGF-β) family of cytokines, which are multifunctional peptides that regulate proliferation, differentiation, adhesion, migration, and functional activation of immune cells. This gene is frequently upregulated in tumor cells, and mutations in the TGF-β1 gene results in human disease [37–39]. TGF- β1 is elevated in the local inflammatory tissue in the intestinal mucosa of UC patients and is associated with development of UC [40, 41].

In recent years, herbal therapy or traditional Chinese medicine for the treatment of patients with UC have shown efficacy in the clinic. Traditional Chinese medicinal enemas can effectively inhibit regional mucosal inflammatory factors and improve disorders associated with immunity [42, 43]. Our results showed that expression of TLR2, NF-κB, IL-1β and TGF-β1 were increased in the intestinal mucosa of UC rats with quantitative PCR analysis. Treatment of UC rats with KJA significantly reduced the expression of NF-κB, IL-1β and TGF-β1in the intestinal mucosa as compared with UC rats treated with normal saline. The expression of KJA on TLR2 was also reduced, although its effect was less dramatic.

Based on our experimental data, we assume that the DNCB combined acetic acid induced pathogenesis of rat UC, led to the destruction of the intestinal mucosa, initiating abnormal changes in the immune system of the intestinal mucosa, and thereby disrupted the balance between local immunity and intestinal flora symbiosis. The disorder between promoting inflammation and suppression of inflammatory factors may lead to an imbalance of immune regulation, causing the abnormal immune response of the intestinal mucosa, which eventually leads to damage of the intestinal mucosa. Application of KJA reverses this imbalanced local immune reaction and may also initiate the recuperation of local intestinal epithelial cells and healing of uncreative lesions. KJA can significantly improve diarrhea, stool and weight loss and other symptoms in rats with UC [23]. Pathological findings showed that KJA can reduce inflammatory cell infiltration, promote ulcer healing with curative effects on UC [22, 23], and thus support the changes in the critical genes we describe here.

The possible mechanism of Uygur medicine KJA to treat ulcerative colitis is probably mediated by reducing the expression of inflammation promoting factors, thus providing potential molecular mechanisms for the curative effect of KJA on UC. Future studies aimed at cell specific analysis of the differentially regulated genes in the colon tissue before and after treatment with KJA may lead to improved understanding of the therapeutic effect of KJA on UC.

## Conclusions

Using the crystal core® 27 rat genome oligonucleotide array, we detected changes in the gene expression profiles from different doses of KJA in a rat model of UC. Through analysis of gene chip data mapped to the KEGG pathways, the effect of KJA in UC rats might be associated with oxidative phosphorylation, disease, metabolism reactions, CAMs, immune responses, and signaling pathways. The quantitative real-time RT-PCR results of TLR2, IL-1β, TGF-β1 and NF-κB expression, verified consistently with the microarray data. On comparison of the Uygur medicine KJA intervention group with the physiological saline negative control group, Uygur medicine KJA can successfully treat UC rats by dampening the expression of TLR2, IL-1β, TGF-β1 and NF-κB. Further studies on differentially expressed genes will help explain the mechanism of action of Uygur medicine KJA.
